# High intercorneal symmetry in corneal biomechanical metrics

**DOI:** 10.1186/s40662-016-0037-7

**Published:** 2016-03-05

**Authors:** XiaoBo Zheng, FangJun Bao, Brendan Geraghty, JinHai Huang, AYong Yu, QinMei Wang

**Affiliations:** The Affiliated Eye Hospital of Wenzhou Medical University, Wenzhou, Zhejiang Province 325027 China; The Institution of Ocular Biomechanics, Eye Hospital, Wenzhou Medical University, No. 270# Xueyuan West Road, Wenzhou City, Zhejiang Province 325027 China; School of Engineering, University of Liverpool, Liverpool, L69 3GH UK

**Keywords:** Intercorneal symmetry, Corneal biomechanical metrics, Corneal stiffness, Ocular response analyzer

## Abstract

**Backgroud:**

To evaluate the symmetry of corneal biomechanical metrics, measured using an ocular response analyzer (ORA) and self-built corneal inflation test platform, in bilateral rabbit corneas and to investigate their relationship with physical intraocular pressure (IOPp).

**Methods:**

Twenty fresh enucleated eyes from ten rabbits were used for ex vivo whole ocular globe inflation. IOP was increased from 7.5 to 37.5 mmHg with 7.5 mmHg steps and biomechanical metrics were acquired using the ORA. At least 3 examinations were performed at each pressure stage. Two biomechanical metrics, corneal hysteresis (CH) and corneal resistance factor (CRF) were recorded and analyzed as a function of IOPp. Corneal specimens were then excised from the intact ocular globe and tested under inflation conditions up to 45.7 mmHg posterior pressure. The experimental pressure-deformation data was analyzed using an inverse modeling procedure to derive the stress-strain behavior of the cornea.

**Results:**

A comparison of corneal shape parameters showed no statistically significant difference (*P* > 0.05) between bilateral eyes. Similarly, there were no statistically significant differences in values of CH, CRF and corneal stiffness (as measured by the tangent modulus, Et) between bilateral eyes (CH: *F* = 0.94, *P* = 0.54; CRF: *F* = 4.42, *P* = 0.35; Et: *F* = 3.15, *P* = 0.12) at different pressure levels. IOPp was highly correlated with CRF while the relationship with CH was less pronounced.

**Conclusions:**

An obvious interocular symmetry in biomechanical metrics is found in this research. IOP has been shown to have important influences on the value of CRF provided by ORA.

## Background

The cornea accounts for about two thirds of the refractive power of the eye and is an important component of the mechanically tough outer ocular membrane [1]. Because of its importance, a great deal of research has been conducted to understand its performance and how it responds to external and internal effects [2, 3]. Careful evaluation of the health and adequacy of the cornea is critical for increasing the accuracy of corneal refractive surgery and ensuring the prevention of iatrogenic keratectasia [4]. The corneal mechanical properties, which are essential for maintaining its dimensional stability and hence clear vision, rely on the cornea’s topography, thickness and the intrinsic properties of the tissue [5].

Refractive surgery has become a common clinical practice that is used to alter the corneal curvature to correct refractive error by controlled cutting of the corneal tissue. Several studies have shown that the biomechanical properties of the cornea play an important role in the final refractive outcome and the predictability of surgical procedures [6, 7]. Biomechanical properties, which have a direct bearing on the corneal structural resistance, can affect the accuracy of intraocular pressure (IOP) measurements [8–10]. Subsequent altered corneal stiffness after ablative corneal refractive surgery can result in erroneous postoperative IOP readings measured by applanation tonometry [5]. Assessment of biomechanics is therefore important for the adequate understanding of corneal behavior in response to mechanical actions, refractive surgery-induced tissue remodeling or non-ablative refractive correction such as conductive keratoplasty (CK), as well as aspects of corneal physiology where mechanics plays a role (e.g. disease, injury, would healing and implants).

It is well established that a degree of symmetry has been observed in fellow eyes of most individuals. The astigmatic axis, IOP, higher-order aberrations, corneal curvature, central corneal thickness and the epithelial thickness, have similar patterns [11–15]. Marked anisometropia is uncommon, either in the magnitude of spherical or astigmatic refractive errors. Corneal biomechanics received much attention in the last few decades, and significant advances have been made in defining corneal hyperelasticity [16, 17], the effects of aging [18, 19], diabetes mellitus [20], hydration [21, 22], estrogen [23] and others. However, little progress has been made with regard to whether or not biomechanical parameters are symmetric in fellow corneas. Therefore, we sought to study the nature of the relationship between the biomechanical material properties in fellow corneas. The confirmation of high interocular symmetry may be helpful in the early assessment of ocular abnormalities, be valuable as a validation of accurate binocular data, and can also assist in the prediction of postoperative outcomes in fellow eyes. However, large asymmetry may warrant repeated clinical measurement of the eyes. Corneal biomechanical measurements are influenced by several factors, such as IOP, corneal swelling and imbibition pressures within the cornea. It is well known that IOP has an important influence on biomechanical metrics of cornea. Consequently, the current study aims to evaluate the interocular symmetry of corneal biomechanical metrics at different pressure levels.

## Methods

### ORA measurement

Ten Japanese white rabbits (2–3 kg) from the Animal Breeding Unit at Wenzhou Medical University were used in this study. All animals were treated in agreement with the ARVO Statement for Use of Animals in Ophthalmic and Vision Research and with the approval of the Animal Care and Ethics Committee of the Eye Hospital, Wenzhou Medical University. The rabbits had their IOP measured using a Tono-pen tonometer (Reichert, Inc., New York, USA) to ensure the eyes were not subjected to elevated IOP. They were euthanized by intravenous injection pentobarbital sodium overdose (Merck, Darmstadt, Germany) according to the weight (100 mg/kg) and the bilateral eyes were immediately enucleated. Both ocular response analyzer (ORA) measurements and the following corneal inflation test were performed at room temperature and within 4 h postmortem to reduce possible effects of post mortem tissue degradation.

An infusion needle was inserted into the vitreous body of the eye through the optic nerve to control the IOP. A transfusion bottle filled with Phosphate Buffered Saline (PBS, Maixin, China) was raised and lowered to change the pressure inside the eye, which was named as physical IOP (IOPp). The pressure was continuously monitored using a pressure transducer (DMP-HS, Hangzhou, China) as described in previous study [24]. The ORA (Reichert, Inc., New York, USA) was used to measure the corneal biomechanical metrics, corneal hysteresis (CH) and corneal resistance factor (CRF), at five different pressure levels (7.5, 15, 22.5, 30 and 37.5 mmHg). Three ORA measurements were taken at each pressure level, and the average value was used for statistical analysis. The interval between three measurements at each manometric level was at least two minutes.

### Corneal inflation testing procedures

After carrying out measurements using the ORA, the corneas were extracted from the eye globes with a 3-mm ring of scleral tissue and all other ocular components were removed. As described previously [25-27], the corneas were mounted onto a custom designed pressure chamber of an inflation test rig using a mechanical clamp and cyano-acrylate glue to provide watertight connection along their ring of scleral tissue. Care was taken to avoid damage to the epithelium and endothelium. The pressure chamber was filled with PBS and connected to a reservoir, whose vertical movement was controlled by a motor to allow smooth increases and decreases in pressure.

The motor attached to the reservoir was controlled by Motion Assistant software (National Instruments Corporation, Texas, US) to set the pressure change rate at 0.427 mmHg/s. All specimens were first subjected to an initial inflation pressure of 3.00 mmHg to ensure a fully inflated and wrinkle-free corneal surface. Loading of up to 45.7 mmHg was applied to the tissue and displacement at the corneal apex was continually monitored using a CCD laser displacement sensor (LK series, Keyence, Milton Keynes, UK) during the tests. The laser beam and pressure transducer were all connected to a personal computer to record the data automatically. The central and peripheral (approximately 1.5 mm away from the limbus) thicknesses of separated cornea were measured using a SP-3000 ultrasonic pachymeter (Tomey Inc, Nagoya, Japan) before the inflation test. Corneal diameters in four directions (horizontal, vertical, and two diagonal directions) were measured using a Vernier caliper. Two further initial corneal shape parameters, corneal radius (R) and shape factor (p) in the temporal-nasal directions, were calculated from the corneal anterior profile data measured by the camera based on the equation: **y**^2^ = − **px**^2^ + 2**rx**. The average value for each specimen was used in the mathematical analysis presented below. Each specimen was tested within 3 h postmortem.

### Inverse modeling

Inverse modelling is a method that can successfully provide material behavior properties from experimental data. It is particularly useful when a simple analytical process is unavailable such as where complex geometric forms exist. Here, this method has been used to derive the gross material properties of the cornea. The design optimization software package LS-OPT (Livermore Software Technology Corp, CA, USA) was used to implement the iterative process. Finite element (FE) solver Abaqus (Dassault Systèmes Simulia Corp., Rhode Island, USA) was used to simulate the response of the cornea model based on the fixed geometric parameters and the adaptable material coefficients. The optimization software adjusted the material coefficients in order to minimize the root mean square (RMS) error between the experimental data and the response of the model; $\mathrm{R}\mathrm{M}\mathrm{S}=\sqrt{\frac{1}{N}{\displaystyle {\sum}_{i=1}^N{\left({\delta}_i^{experimental}-{\delta}_i^{numerical}\right)}^2}}$, where *N* is the number of pressure levels = 25, and *δ* the apical rise at a particular pressure, *i*.

Twenty FE models were developed representing the bilateral corneas. The models contained unique geometry, which adopted the thickness measurements taken at the cornea center and at four peripheral points, the curvature and shape factor measurements made in the nasal-temporal direction in addition to the limbal diameter measurements. Each model was constructed from 4071, 15-noded continuum elements arranged in twelve rings and one element layer representing the stroma. They also assumed encastre connection along the limbus to simulate connection to the mechanical clamps.

The global response of the models and cornea were assessed at the singularity of the pressure-apical rise measurement and no measurements of anisotropic or viscoelastic behavior was conducted. The material behavior of the cornea was therefore represented by a first order hyperelastic Ogden model with the strain energy density function:

1 $$ \mathrm{W}=\frac{2\mu }{a^2}\left({{\overline{\lambda}}_1}^{\alpha }+{{\overline{\lambda}}_2}^{\alpha }+{{\overline{\lambda}}_3}^{\alpha }-3\right)+\frac{1}{D}{\left(J-1\right)}^2 $$

where W is the strain energy per unit volume, ${\overline{\lambda}}_k$ the deviatoric principal stretches = J^-1/3^× *λ*_*k*_ (k = 1, 2, 3), λ_1_, λ_2_, λ_3_ the principal stretches, *J* = λ_1_λ_2_λ_3_. *α* and *μ* are the material parameters denoting the strain hardening exponent and the shear modulus, respectively. *D* is a compressibility parameter, whose value is dependent on Poisson’s ratio, ν; $\mathrm{D}=\frac{3\left(1-2\nu \right)}{\mu \left(1+\nu \right)}$. Previous studies have estimated values for Poisson’s ratio for ocular components, including the cornea, which ranges between 0.45 and 0.5, denoting a near incompressible behavior [28-29]. Here, corneal tissue was assumed to be incompressible. The other material coefficients, *μ* and *α*, were obtained from the inverse modeling procedure described above. Their values were allowed to vary between 0.0001 and 1.0, and between 20 and 350, respectively with baseline values 0.04 and 120, which were suitable for all specimens. The use of a first order material model was found sufficient in earlier studies [30]. This reduced computation time during the inverse modeling procedure by reducing the number of variables requiring optimization.

### Statistical analysis

All analyses were performed using the PASW Statistics 20.0 software (SPSS Inc., Chicago, USA). Comparison of corneal shape parameters and biomechanical metrics was performed using paired *T* test and Hotelling T-Square Test, respectively. In this study, P-values of less than 0.05 were considered to be statistically significant. A second-order polynomial regression was used to explore the nonlinear correlation between IOPp and biomechanical metrics and their interocular differences.

## Results

### ORA parameters

There were no statistically significant differences between fellow eyes in corneal radius, shape factor, corneal diameter or central and peripheral corneal thickness (Table 1). The differences of CH and CRF among the five pressure levels between fellow eyes (Table 2) were not statistically significant (CH: *F* = 0.94, *P* = 0.54; CRF: *F* = 4.42, *P* = 0.35). The correlation analysis of IOPp with the two biomechanical metrics CH and CRF was based on a second-order polynomial regression. IOPp was weakly correlated with CH (R^2^ = 0.09, 0.03) while the relationship with CRF was more pronounced (R^2^ = 0.84, 0.83, for right and left eyes, respectively). The correlations of interocular differences of CH and CRF with IOPp were extremely low (R^2^ = −0.02, R^2^ = −0.01).

**Table 1:** Average and standard deviation values of corneal shape parameters between right and left eyes

Ocular parameters	Right eyes	Left eyes	t	P
R (mm)	5.9 ± 0.89	5.97 ± 0.92	−0.23	0.83
p	0.59 ± 0.36	0.6 ± 0.37	−0.13	0.90
CCT (μm)	360.7 ± 12.1	358.7 ± 15.7	0.32	0.76
PCT (μm)	356.3 ± 13.4	351.4 ± 15.2	1.06	0.32
CD (mm)	12.87 ± 0.47	12.98 ± 0.47	−1.87	0.10

*R*= corneal radius, *p= * corneal shape factor, *CCT=* central corneal thickness, *PCT=* peripheral corneal thickness, *CD=* corneal diameter

**Table 2:** Average and standard deviation values of CH and CRF between right and left eyes at different pressure levels

Biomechanical parameters	Pressure	Right eye	Left eye	Interocular differences
CH	7.5 mmHg	4.07 ± 1.07	3.83 ± 0.63	0.24 ± 1.39
	15.0 mmHg	5.02 ± 1.12	5.05 ± 0.97	−0.04 ± 1.72
	22.5 mmHg	5.41 ± 1.92	4.87 ± 1.46	0.54 ± 2.4
	30.0 mmHg	5.68 ± 1.45	4.95 ± 2.16	0.73 ± 2.41
	37.5 mmHg	5.86 ± 2.77	4.56 ± 1.80	1.02 ± 3.25
CRF	7.5 mmHg	0.79 ± 0.74	0.71 ± 0.56	0.31 ± 0.79
	15.0 mmHg	3.38 ± 1.02	3.12 ± 0.74	0.32 ± 1.42
	22.5 mmHg	5.30 ± 1.54	5.01 ± 1.09	0.28 ± 1.59
	30.0 mmHg	7.81 ± 1.30	7.11 ± 1.80	0.7 ± 1.68
	37.5 mmHg	10.01 ± 2.01	8.64 ± 1.70	1.11 ± 2.39

CH= corneal hysteresis, CRF= corneal resistance factor

### Experimental behavior and material constitutive models

**Fig. 1:**
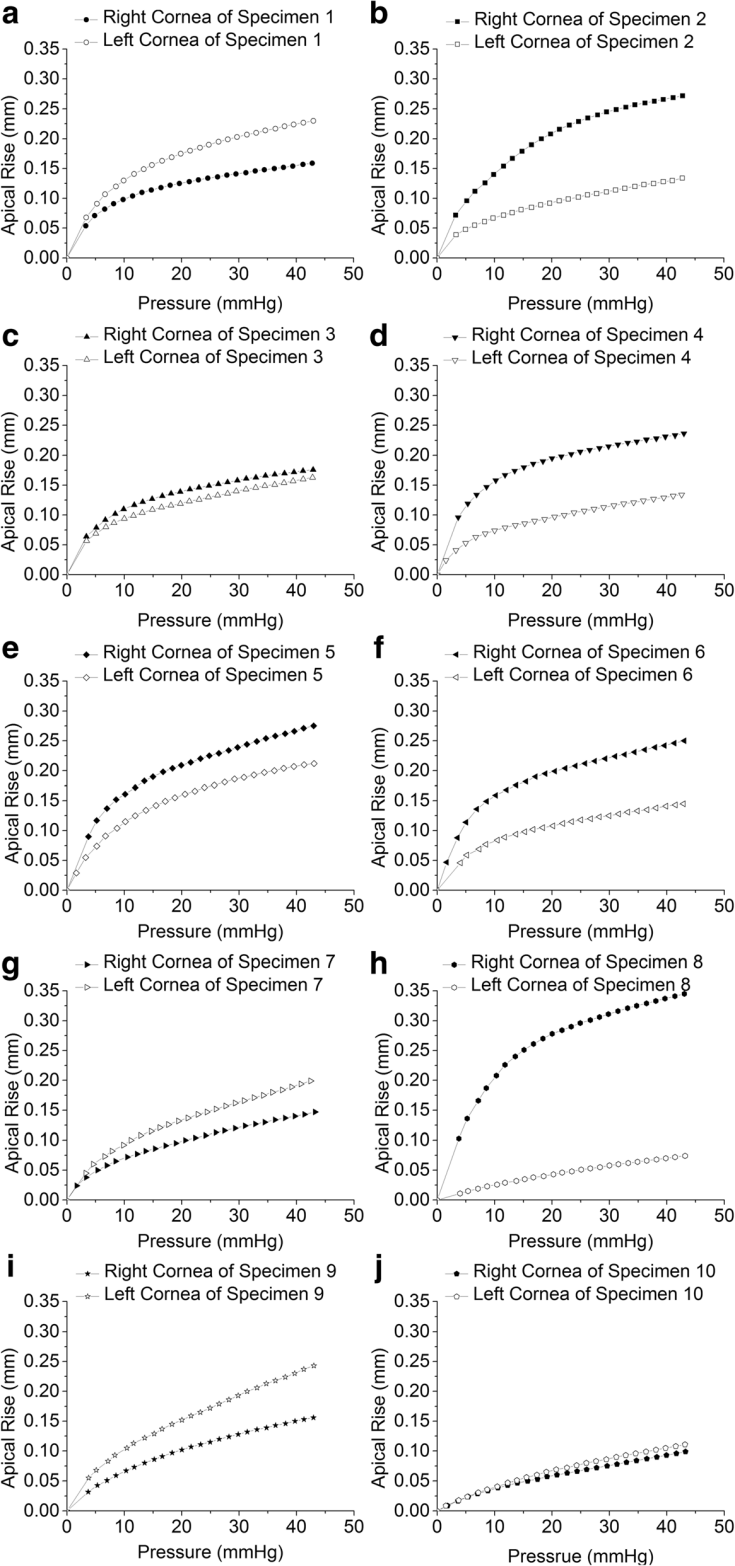
Pressure-rise behavior of corneal specimens between right and left eyes, "**a**-**j**" means specimen 1 to specimen 10

**Fig. 2:**
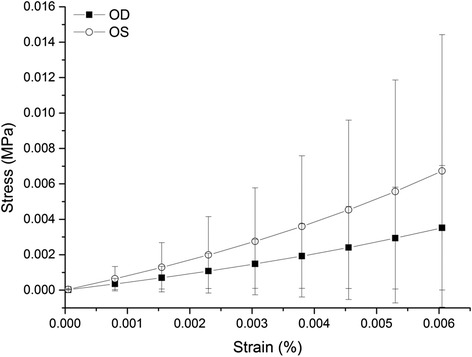
Comparison of average stress-strain behavior between right and left eyes. The error bars represent the standard deviation of stress values

The pressure-apical rise behaviors of all specimens, as obtained in the fourth loading cycle, are compared in Fig. 1. In all cases, specimens exhibited an initial low stiffness and a final, considerably higher stiffness (as measured by the tangent to the pressure-displacement curve). Analysis of the interocular pressure-displacement relationships reveals a slightly different trend in behavior between fellow eyes. The inverse modeling process described above was used to obtain values for parameters *α* and *μ*, and therefore derive a constitutive model for each cornea that provides the best possible fit (lowest RMS) with the experimentally obtained pressure-displacement results (Table 3). Figure 2 further presented the stress-strain behavior as obtained from the inverse modeling exercise, along with a comparison between two eyes showing a slight difference of bilateral sides. The stress-strain constitutive models also enabled the determination of the tangent modulus (a measure of material stiffness) at different stress levels: Et = dσ/dε ≈ ∆σ/∆ε. In spite of the nonlinear form of the stress-strain results, the relationship between the tangent modulus (Et) and the stress (σ) was expected to be close to linear [31, 32]. In this study, the Et-σ relationship was assessed for all specimens. In order to quantify this effect, five stress levels (0.001, 0.002, 0.003, 0.004 and 0.005 MPa) were selected for analysis and comparison. The five stresses were equivalent to pressures of approximately 7.5, 15, 22.5, 30 and 37.5 mmHg, respectively, that represent low to high levels of IOP in rabbits and humans. Differences in Et between bilateral eyes were not statistically significant (*F* = 3.15, *P* = 0.12) and the correlations of interocular differences of Et with IOPp were low (R^2^ = −0.04).

**Table 3:** Average and standard deviation values of tangent modulus between right and left eyes at different stress levels

Biomechanical parameters	Stress (MPa)	Right eye	Left eye	Interocular differences
Tangent Modulus	0.001	0.5 ± 0.39	0.86 ± 0.83	−0.36 ± 0.87
	0.002	0.6 ± 0.39	0.97 ± 0.82	−0.37 ± 0.89
	0.003	0.71 ± 0.4	1.09 ± 0.82	−0.38 ± 0.92
	0.004	0.82 ± 0.42	1.21 ± 0.83	−0.39 ± 0.95
	0.005	0.94 ± 0.43	1.34 ± 0.83	−0.41 ± 0.98

## Discussion

For assessment of the health and adequacy of corneal tissue for corneal procedures, corneal biomechanical properties can be used to screen patients for conditions including mild keratoconus or form fruste keratoconus, pellucid marginal degeneration and other ectasias. These parameters may allow earlier detection of keratoconus or identify those patients at risk for developing mild and marked keratectasia post LASIK or photorefractive keratectomy (PRK). Corneal biomechanical properties, in addition to being obtained for corneal refractive surgery, may become a critical test in glaucoma evaluation. Clinically, it may be valuable to use high symmetry in routine clinical assessments as a validation of accurate binocular data so that the absence of high symmetry may warrant repeat measurement of the eyes. An additional clinical use of this reported high symmetry in biomechanical properties is in IOP calculations for a post laser-assisted in situ keratomileusis (LASIK) eye if only one eye was treated and preoperative data was unavailable.

The ORA, one of the most commonly used tonometers, has become a popular clinical device for evaluating biomechanical properties since its launch in 2005 [33]. Although CH and CRF can be influenced by many factors, such as corneal health state, corneal thickness, IOP and the properties of the whole eye [33-37], they are widely accepted as an important factor in understanding the biomechanical state of the cornea and clinical diagnosis of eye diseases. Both parameters have been found to display symmetry in bilateral eyes as shown in Montard and Shen’s research [38, 39]. To our knowledge, this study is the first attempt to statistically analyze the symmetry of the nonlinear corneal biomechanical properties between the right and left eyes. The non-statistically significant differences of CH and CRF remained stable among the five pressure levels between fellow eyes and demonstrated symmetrical nonlinear corneal biomechanical properties. Similar to an earlier study by Roberts [40], the present results showed that eyes displayed variable biomechanical metrics with different values of IOP (Table 2). Although no statistically significant differences were observed in CH and CRF for bilateral rabbit eyes during the current study, a previous study by Tejwani et al. [41] reported differences in human eyes. However, ORA metrics were measured at exactly the same IOP levels for both eyes in the current study while there was a 1.2 mmHg difference between IOPcc for left and right eyes in Tejwani’s study. It is now known that IOP can have important influences on most corneal biomechanical metrics provided by the ORA, as mentioned in our previous research [24]. Therefore, it is possible that this IOP difference may have contributed to the observed differences in the ORA metrics reported by Tejwani et al.

CH had a weak correlation with IOP whereas a strong positive correlation was found between CRF and IOP. However, the results presented herein were different from other studies [42–44]. This may be caused by differences in pressure measurement methods, specimen species and the type of mount used in the experimental set up. For instance, the current study directly measured IOP using a pressure transducer whereas transcorneal pressure was measured in other studies [42–44]. These studies also used anterior chamber mounts while a whole-globe mount was used in the current study. Consequently, the measurements obtained from the ORA may be affected by the scleral stiffness in addition to that of the cornea. Furthermore, the collagen orientation in rabbit and porcine corneas has been shown to differ from that of human’s [45], which may result in altered mechanical response.

Although various approaches have been designed to measure corneal biomechanical properties in vivo [33, 46], efforts to determine the tangent modulus still rely on ex vivo experiments involving human and in some cases animal corneas. The use of animal corneas [25, 47] (e.g. rabbit and pig) as an approximate model for human corneas in mechanical property characterization were particularly important in light of the need to acquire statistically significant material property data, which is extremely difficult to obtain from human donor corneas. A number of studies on the mechanics of the cornea, both intact and cut into strips, have been published which confirmed its viscoelastic, nonlinear material behavior [48, 49]. The stress strain relationship can be divided into two distinctive phases: the matrix regulated phase with low stiffness and collagen regulated phase with much higher stiffness [50]. The fundamental biomechanical parameter, tangent modulus, is defined as the tangent ratio of the stress and strain relationship and is not a constant. Also, it is shown that high and stable interocular corneal symmetry exist in the tangent modulus of normal corneas at different stress levels. Compilation of normative data on corneal tangent modulus may assist in detecting early keratoconus.

The values of CH and CRF in left eyes were lower than those observed in right eyes whereas Et was higher in the left eyes when compared to right eyes. However, the differences were not statistically significant and may be due to the small sample size or influenced by noise. Interocular biomechanical differences increased as IOP was increased, a finding that could be linked with corneal edema [51]. At an IOP of 40 mmHg and a temperature around 15 °C, the corneal imbibition pressure may reduce and draw water into the corneal stroma [52, 53]. The resulting increase in water content can change corneal biomechanical properties without apparent morphological effects [53]. Since the “border pressure” inducing this phenomenon is slightly different for each cornea, the effect caused by the increasing IOP can vary for corneas and induce increased differences between eyes at increased pressure levels.

## Conclusions

In conclusion, although a large range of values exists in CH, CRF and the corneal tangent modulus, there was a very high interocular symmetry between right and left normal corneas. Further studies may disclose how well variations in this high symmetry could be used to predict abnormalities of the cornea including post postoperative keratectasia and quality of visual outcomes after corneal refractive surgery. Moreover, the results of the current study could be useful in aiding the development of new technologies that aim to eliminate the effect of corneal biomechanical properties on transcorneal IOP measurements. These results may also provide useful information that could help evaluate the status of the corneal endothelial barrier and hence the endothelial cells that play an important role in imbibition pressure maintenance and the prevention of corneal swelling. Due to the difficulty in obtaining sufficiently large numbers of human eyes for research purposes, the current study has attempted to address these issues using rabbit eyes due to their similarity to human eyes. Nevertheless, validation of the results in human eyes will be essential before translating into clinical understanding and practice.
